# Transplantation Induces Profound Changes in the Transcriptional Asset of Hematopoietic Stem Cells: Identification of Specific Signatures Using Machine Learning Techniques

**DOI:** 10.3390/jcm9061670

**Published:** 2020-06-01

**Authors:** Daniela Cilloni, Jessica Petiti, Valentina Campia, Marina Podestà, Margherita Squillario, Nuria Montserrat, Alice Bertaina, Federica Sabatini, Sonia Carturan, Massimo Berger, Francesco Saglio, Giuseppe Bandini, Francesca Bonifazi, Franca Fagioli, Lorenzo Moretta, Giuseppe Saglio, Alessandro Verri, Annalisa Barla, Franco Locatelli, Francesco Frassoni

**Affiliations:** 1 Department of Clinical and Biological Sciences, University of Turin, 10043 Turin, Italy; jessica.petiti@unito.it (J.P.); valentina.campia@izsto.it (V.C.); s.carturan@sanluigi.piemonte.it (S.C.); giuseppe.saglio@unito.it (G.S.); 2 Department of Pediatric Hemato-Oncology and Stem Cell and Cellular Therapy Laboratory, Institute G. Gaslini, Largo G Gaslini, 16147 Genova, Italy; MarinaPodesta@ospedale-gaslini.ge.it (M.P.); FedericaSabatini@gaslini.org (F.S.); 3 Department of Informatics, Bioengineering, Robotics and Systems Engineering, University of Genoa, via Dodecaneso 35, 16146 Genoa, Italy; margherita.squillario@unige.it (M.S.); alessandro.verri@unige.it (A.V.); annalisa.barla@unige.it (A.B.); 4 Pluripotent stem cells and activation of endogenous tissue programs for organ regeneration (PR Lab), Institute for Bioengineering of Catalonia (IBEC) c/Baldiri Reixac 15–21, 08028 Barcelona, Spain; nmontserrat@ibecbarcelona.eu; 5 CIBER of Bioengineering, Biomaterials and Nanomedicine (CIBER-BBN), 28029 Barcelona, Spain; 6 Department of Pediatric Hematology and Oncology IRCCS; Ospedale Pediatrico Bambino Gesù, Rome, University of Pavia, 27100 Pavia, Italy; aliceb1@stanford.edu (A.B.); franco.locatelli@opbg.net (F.L.); 7 Pediatric Onco-Hematology, Stem Cell Transplantation and Cellular Therapy Divisions, Regina Margherita Children Hospital, 10126 Turin, Italy; massimo.berger@unito.it (M.B.); francesco.saglio@unito.it (F.S.); franca.fagioli@unito.it (F.F.); 8 Azienda Universitaria Ospedaliera di Bologna, Policlinico S. Orsola-Malpighi, 40138 Bologna, Italy; giuseppe.bandini@unibo.it (G.B.); francesca.bonifazi@unibo.it (F.B.); 9 Department of Immunology, IRCCS Bambino Gesù Children’s Hospital, 00165 Rome, Italy; lorenzo.moretta@opbg.net

**Keywords:** hematopoietic stem/progenitor cell, cord blood, stem cell transplantation

## Abstract

During the phase of proliferation needed for hematopoietic reconstitution following transplantation, hematopoietic stem/progenitor cells (HSPC) must express genes involved in stem cell self-renewal. We investigated the expression of genes relevant for self-renewal and expansion of HSPC (operationally defined as CD34+ cells) in steady state and after transplantation. Specifically, we evaluated the expression of ninety-one genes that were analyzed by real-time PCR in CD34+ cells isolated from (i) 12 samples from umbilical cord blood (UCB); (ii) 15 samples from bone marrow healthy donors; (iii) 13 samples from bone marrow after umbilical cord blood transplant (UCBT); and (iv) 29 samples from patients after transplantation with adult hematopoietic cells. The results show that transplanted CD34+ cells from adult cells acquire an asset very different from transplanted CD34+ cells from cord blood. Multivariate machine learning analysis (MMLA) showed that four specific gene signatures can be obtained by comparing the four types of CD34+ cells. In several, but not all cases, transplanted HSPC from UCB overexpress reprogramming genes. However, these remarkable changes do not alter the commitment to hematopoietic lineage. Overall, these results reveal undisclosed aspects of transplantation biology.

## 1. Introduction

Hematopoietic stem cell transplantation (HSCT) has cured hundreds of thousands of human beings affected by malignant and non-malignant disorders [1]. A key element of its feasibility and success relies on the redundancy of hematopoietic stem cells (HSCs) in the bone marrow (BM) that allows 1% of donor BM cells to regenerate a new hematopoietic system in a short period. Similar results are obtained by transplanting umbilical cord blood (UCB) cells.

The BM cellularity is estimated to be approximately 10^12^ cells; thus, it can be extrapolated that CD34+ cells expand approximately by a factor of 2 logs after bone marrow transplant (BMT) and 3 logs after umbilical cord blood transplant (UCBT). Because the impact of seeding efficiency should also be taken into account [2] to calculate these figures, the magnitude of this expansion is probably higher. It has been argued that HSC might undergo some sort of exhaustion after transplantation as shown by serial transplantation in mice [3] and by transplants in humans where the frequency of long-term culture-initiating cells (LTC-IC) is permanently reduced [4]. However, Iscove and Nawa [5] have elegantly disputed this concept. Curiously, it was shown that, in children, the reconstitution of the HSC reservoir was superior after UCBT than after adult HSCT, notwithstanding both neutrophil and platelet recovery is delayed after UCBT [6]. Thus, UCB HSCs seem to display very efficient self-renewal machinery. Altogether, the previous considerations depict a rather complex scenario.

To investigate how HSC reorganize their transcriptional asset to cope with the need for hematopoietic regeneration, we evaluated the expression of 91 genes selected for their role in self-renewal and stemness maintenance. We evaluated the transcriptional asset in CD34+ cells obtained from baseline normal BM cell aspiration or UCB units and from patients transplanted with either adult BM/mobilized peripheral blood (PB) or UCB cells. Thus, we investigated the self-renewal program of hematopoietic stem/progenitor cells (HSPC) of different origins by using different donor/recipient combinations. First, an exploratory analysis was performed to disclose a set of genes significantly upregulated in transplanted CD34+ cells. Then, a multivariate sparsity-inducing machine learning algorithm was used to identify four gene signatures with predictive capabilities. Furthermore, the four signatures underwent a functional characterization that identified a set of the Kyoto Encyclopedia of Genes and Genomes (KEGG) pathways as well as an inferred network of gene associations.

## 2. Experimental Section

### 2.1. Cells Sources

The study was approved by the local ethics committee San Luigi Gonzaga (number of approval 230/CEI). All patients signed written informed consent. All experiments were performed in accordance with the relevant guidelines and regulations. CD34+ cells were enriched from 12 umbilical cord blood (UCB) units and 15 adult donors (8 Granulocyte-colony stimulating factor (G-CSF)-mobilized peripheral blood stem cells (PBSC) and 7 BM harvests). In addition, CD34+ cells were enriched from BM collected at different time points after HSCT in the following combinations of stem cell source/recipient: 13 adult patients transplanted with single UCB unit via intra-bone [7], 29 patients (5 adults and 24 pediatric) transplanted with adult HSPCs. We established that within the HSCT group the gene expression distribution in the adult patients was not statistically different from that calculated in the pediatric patients based on the result of a paired *t*-test that showed a *t*-test value = 0.6716, with a degree of freedom = 90 and critical value 1.987. We, therefore, gathered adult and pediatric patients. The clinical characteristics of patients are summarized in Supplementary Table S1. Additional CD34+ cells were separated from 10 UCB, 9 PBSC from adult donors, 15 BM of adults after UCBT, and 10 BM samples from patients after HSCT from adult donors. These 44 samples were analyzed at the protein level only. Patient samples after transplantation were all collected in patients with full donor chimerism and without any evidence of disease based on routine diagnostic analysis.

### 2.2. Induced Pluripotent Stem Cell (iPS) Line Maintenance

Human iPS cell lines (kindly provided by Dr. Niels Geijsen, Hubrecht Institute, Utrecht, the Netherlands) were derived from human skin fibroblasts by transducing OCT4, KLFA, SOX2, and c-MYC transgenes using lentiviral vectors as described [8]. Additional experiments were carried out on human iPS lines obtained as previously described [9].

### 2.3. CD34+ Cell Enrichment

Mononuclear cells (MNC) were isolated from BM, PBSC, and UCB and CD34+ cells enriched by magnetic immune-selection (Miltenyi Biotech, Bergisch Gladbach, Germany), according to the manufacturer’s protocol. Only samples with CD34+ cell purity ranging from 90% to 95% were used.

### 2.4. Real-Time Quantitative (RQ)-PCR Analysis by Low-Density Array/Microfluidic Card

Total RNA was extracted using TRIzol and RNAqueous^®^-Micro Kit (Ambion, Life Technologies, Carlsbad, CA, USA), and cDNA was synthesized by RQ-PCR using High Capacity cDNA Reverse Transcription kit (Applied Biosystems, Life Technologies, Carlsbad, CA). For the study, 384 wells TaqMan Low-Density Array or MicroFluidic Card (MFC) (Applied Biosystems, Life Technologies, Carlsbad, CA) were used. Each well contains lyophilized specific primers and probes for target genes. The expression levels of 91 genes and three different housekeeping genes, *GUSB*, *rRNA18S*, and *ABL*, were investigated. A list of the examined genes and their main functions is provided in Supplementary Table S2. The analysis was performed in duplicate and results analyzed by SDS2.3 (ABI prism 7900 HT Fast, Life Technologies, Carlsbad, CA) software. Universal Reference RNA (Stratagene, La Jolla, CA, USA) was used as calibrator. Raw data were normalized against endogenous control gene (*GUSB*) and then compared to the calibrator.

### 2.5. Immunofluorescence Staining

CD34+ cells were fixed as previously described [10]. A complete list of the antibodies used is available in Supplementary Table S2. As secondary antibodies, Alexa Fluor 488-labeled goat anti-rabbit IgG (cat. A11008), goat anti-mouse IgG, and donkey anti-goat IgG (Invitrogen Life Technologies, Carlsbad, CA, USA) were used in a dilution of 1:1000. Cells were analyzed under a fluorescence microscope (Leica DM2000 LED, Leica Microsystem, Wetzlar, Germany) and images acquired using Leica application Suite 4.4.0 software and quantified using Image J.

### 2.6. Sparse Multivariate Analysis

l_1_l_2FS_ is an embedded regularization method for variable selection capable to identify subsets of discriminative genes. The algorithm can be tuned to give a minimal set of discriminative genes or larger sets including correlated genes. The method is based on the elastic net optimization principle presented by Zou [11] and further developed by De Mol et al. [12] and successfully applied in the analysis of molecular high-throughput data [13,14,15,16]. First, we fix some notation and then we explain the idea behind the algorithm, referring to De Mol et al. [13] for a detailed description of the method. Assume we are given a collection of n samples, each represented by a d-dimensional vector x of measurements (e.g., the gene expressions). Each sample is also associated with a binary label y, assigning it to a class (e.g., UCB or UCBT). The dataset is, therefore, represented by an n × d matrix X and Y is the n-dimensional labels vector. Using only a subset of the given data (training set), the l_1_l_2FS_ algorithm looks for a linear function f(x) = β*∙x, whose sign gives the classification rule that can be used to associate a sample to one of the two classes. The classification performance of f(x) is then assessed on the remaining samples (test set) that were not used to build the model function. Note that the vector of weights β* is forced to be a sparse vector, which is some of its entries are zero, then some variables will not contribute to building the estimator f(x). The weight vector β* is found in the so-called model selection phase, which consists in selecting the optimal values for two regularization parameters denoted with τ* and λ*, respectively. Model selection and classification accuracy assessment are performed within two nested K-fold cross-validation loops, similarly to Barla et al. [17], in order to guarantee an unbiased result. As a consequence of the external loop of cross-validation, l_1_l_2FS_ provides a set of K lists of discriminant variables; therefore, it is necessary to choose an appropriate criterion [17] in order to assess a common list of relevant variables. We based ours on the absolute frequency, i.e., we decided to promote as relevant variables the most stable genes across the lists. The threshold we used to select the final lists was chosen according to the slope variation of the number of selected genes vs. frequency, with its value being 50%. In this way, we managed to cut out those variables that were not stable across the cross-validation lists.

### 2.7. Performance Metrics

We evaluated the prediction performance through the accuracy and the Matthews Correlation Coefficient (MCC) metrics. In customary notation, when considering a classification task, a classifier assigns the considered samples to two possible classes: Negative (−1) and positive (+ 1). In this case, the true positives (TP) are the positive examples correctly classified as +1, the true negatives (TN) are the negative examples classified as −1, the false negatives or Type II error (FN) are negative examples misclassified as members of the positive class and, similarly, the false positives or Type I error (FP) is the negative example wrongfully assigned to class +1. Accuracy is used as a statistical measure of how well a binary classification test correctly identifies or excludes a condition. In other words, accuracy is the proportion of true results (both TP and TN) among the total number of cases examined. Accuracy ranges from 0% to 100%, which is the perfect classification. A random classifier would achieve an accuracy rate based on the prevalence of the two classes. If the prevalence is the same, i.e., the amount of samples is equal in the two classes, a random classifier would achieve 50% accuracy. MCC is a metric that takes into account all the parameters just defined above and it is defined as:

(1) MCC=(TP×TN−FP×FN)/√((TP+FP)(TP+FN)(TN+FP)(TN+FN))

MCC, unaffected by the presence of unbalanced classes, ranges between −1 and +1. The greater MCC the better the prediction with negative score marking below random performance.

### 2.8. Network Inference and Identification of Gene Function

In order to verify if the identified genes belonging to the respective signatures were functionally associated, we used the Search Tool for Recurring Instances of Neighbouring Genes (STRING), a publicly available web server able to find a set of potentially functionally associated genes to a gene query list [18]. The results of this functional characterization analysis, underline if the identified genes within each signature are connected through eight possible types of edge connections (Conserved neighborhood, Gene Fusions, Phylogenetic co-occurrence, Co-expression, Database imports, Large-scale experiments, Literature co-occurrence, Combined score), represented in different colors in the corresponding plots. For the functional analysis of the gene signatures, we also used the online gene set enrichment analysis toolkit WebGestalt [19]. The toolkit performs the functional characterization by a gene set enrichment analysis in several databases including Gene Ontology [20] and the Kyoto Encyclopedia of Genes and Genomes (KEGG) [21]. Given a KEGG pathway and a reference set (such as the entire human genome), the enrichment is based on the comparison between the fraction of signature genes in the pathway and the fraction of pathway genes in the reference set. The signature is enriched in the KEGG pathway if the former is larger than the latter fraction. To perform the enrichment analysis in KEGG, we selected the WebGestalt human genome as reference set, *p*–value ≤ 0.05 as level of significance, 3 as the minimum number of genes, and the default Hypergeometric test as statistical method.

## 3. Results

### 3.1. Gene Expression Analysis

The analyzed dataset comes from RQ-PCR experiments in which the relative expression of 91 genes (see Supplementary Table S2 for a complete list of genes and their function) was measured in CD34+ cells enriched from 12 umbilical cord blood (UCB) units and 15 adult donors (8 G-CSF-mobilized peripheral blood stem cell (PBSC) and 7 BM harvests). In addition, CD34+ cells were enriched from BM collected at different time points after HSCT in 13 adult patients transplanted with single-unit UCB via intra-bone according to a previously published protocol [7] and in 29 patients (24 pediatric and 5 adults) transplanted with adult HSCs. Our RQ-PCR does not provide absolute quantification of gene expression but a relative measurement compared to a standard calibrator. The clinical characteristics of patients are summarized in Supplementary Table S1. We are aware that CD34+ cell subset represents a heterogeneous cell population; however, this did not prevent the identification of very reproducible gene expression clusters characterizing specific cell populations. Thus, throughout the manuscript, the term HSPC is operationally used to indicate CD34+ cells. The pairwise comparison among groups allowed identifying the most relevant genes and pathways discriminating them. Throughout the paper, we identified several gene signatures by means of differential expression analysis and multivariate regularization analysis. For the former, we used standard Mann–Whitney test, while for the latter we used l_1_l_2FS_, a regularization method enforcing sparse solution [13] and set in a nested cross-validation structure to ensure reproducibility and robustness [17]. This method was proven to be very effective for high-throughput molecular data analysis [14,16,22]. We pooled together both mobilized peripheral blood stem cells (PBSC) and bone marrow stem cells (BMSC), referring to the pooled group as adult HSPC, as no significant differences appeared when comparing the two subgroups. Similarly, the comparison between the gene expression profile in adult and pediatric patients after HSCT failed to demonstrate relevant differences, therefore, we gathered the two groups.

#### 3.1.1. CD34+ Cells after UCBT Express Genes of Self-Renewal, Stem Cell Maintenance, and Reprogramming

**Figure 1:**
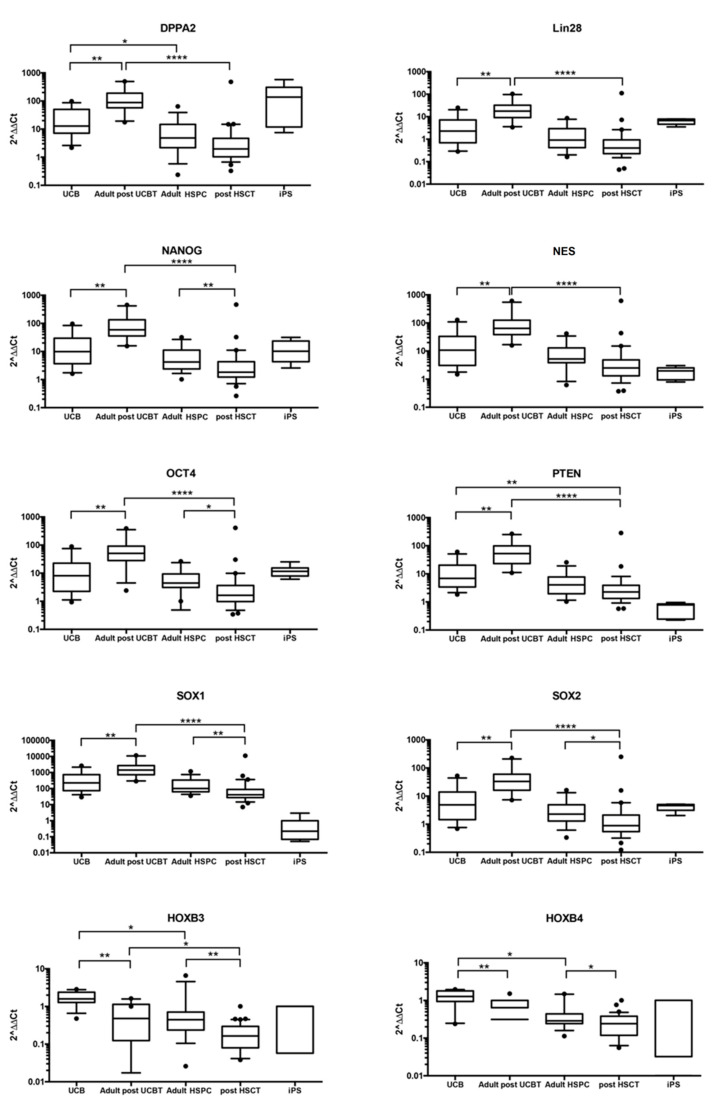
Gene expression analysis showed a different expression of *DPPA2, LIN28, NANOG, NES, OCT4, PTEN, SOX1, SOX2, HOXB3,* and *HOXB4* in the 5 groups of samples evaluated. mRNA expression levels are expressed as 2^−∆∆Ct^ in CD34+ cells separated from: umbilical cord blood (UCB) units, bone marrow (BM) cells from adult patients after UCB transplant (UCBT), from adult healthy donors (adult hematopoietic stem/progenitor cells (HSPC)), from BM cells from adult and pediatric patients after adult hematopoietic stem cell (HSC) transplant (post-HSCT) and iPS. Horizontal bars indicate the median value. Figure showed that *DPPA2, LIN28, NANOG, NES, OCT4, PTEN, SOX1,* and *SOX2* were upregulated in UCBT compared to UCB group. In contrast, these genes were downmodulated in post HSCT compared to adult HSPC. *HOXB3* and *HOXB4* showed downregulation in both transplanted group (UCBT and HSCT) compared to UCB and adult HSPC, respectively. Except for *OCT4, HOXB3,* and *HOXB4,* less expressed in adult HSPC compared to UCB, no significant differences were found between the two groups. (* *p* ≤ 0.05; ** *p* ≤ 0.01; **** *p* ≤ 0.0001).

Because our previous studies of HSPC cell biology suggested that self-renewal of HSPC after UCBT was more efficient than after adult HSC transplant [6], we started to analyze the genes that were expressed in CD34+ cells after UCBT compared to CD34+ cells from UCB. Among 91 genes tested, Mann–Whitney test allowed to identify 10 top genes differentially expressed in UCB CD34+ cells before and after transplant. These 10 genes were selected for the high difference in expression between CD34+ cells from UCB units and CD34+ cells after UCBT regardless of the level of expression. In detail, *DPPA2*, *LIN28*, *NANOG*, *NES*, *OCT4*, *PTEN*, *SOX1*, and *SOX2* were significantly upregulated in CD34+ after Cord Blood Transplantation compared to CD34+ obtained from Cord Blood Units (*p* < 0.01) (Figure 1). Most of these genes are known to play a key role in reprogramming somatic cells and are used in different combinations to generate iPS starting from somatic cells [8,9,23] (see Supplementary Table S2). By contrast, *HOXB3* and *HOXB4* appeared downregulated (*p* < 0.01) (Figure 1). As shown in Figure 1, we further extended the analysis by performing a comparison between: Adult donor CD34+ cells vs. adult and pediatric CD34+ cells after HSCT, CD34+ cells from UCB vs. adult CD34+ cells, and adult CD34+ cells after UCBT vs. adult and pediatric CD34+ cells after adult HSCT.

#### 3.1.2. Self-Renewal, Stem Cell Maintenance, and Reprogramming Genes Are not Differentially Expressed in CD34+ Cells from UCB vs. Adult CD34+ Cells

The pattern of expression of *LIN28*, *NANOG*, *NES*, *OCT4*, *PTEN*, *SOX1*, and *SOX2* was similar in UCB and adult HSPC. Only the expression level of *DPPA2*, *HOXB3*, and *HOXB4* was significantly decreased in adult HSPC compared to UCB (*p* < 0.05). Gene expression analysis showed a different expression of *NANOG*, *OCT4*, *SOX1*, *SOX2*, *HOXB3*, and *HOXB4* after transplantation with adult HSPC. A significant decrease in expression of *NANOG*, *SOX1*, and *HOXB3* (*p* < 0.01) and of *OCT4*, *SOX2*, and *HOXB4* (*p* < 0.05) was observed in CD34+ cells from patients transplanted with adult hematopoietic cells when compared with CD34+ cells from adult donors. There was a trend of reduction in the expression levels of *DPPA2*, *LIN28*, *NES*, and *PTEN*, although not statistically significant (Figure 1).

#### 3.1.3. Genes Regulating Self-Renewal, Cell Reprogramming, and Stem Cell Maintenance Are Overexpressed in CD34+ Cells after UCBT but not after Adult HSCT

Interestingly, adult patients transplanted with UCB showed significantly higher gene expression values of *DPPA2*, *LIN28*, *NANOG*, *NES*, *OCT4*, *PTEN*, *SOX1*, *SOX2* (*p* < 0.0001), and *HOXB3* (*p* < 0.05) compared to adult patients transplanted with adult HSPC. However, these values were not statistically significant, and the median value of *HOXB4* was lower after adult HSCT than after UCBT. 

#### 3.1.4. Some Reprogramming Genes Are Similarly Expressed in CD34+ Cells after UCBT and iPS Although Their Overall Picture of Gene Expression Is Divergent

Because we observed that CD34+ cells after UCBT overexpress genes involved in somatic cell reprogramming, we reasoned that a comparison with the expression of the same 91 genes in iPS cells was necessary. As shown in Figure 1, the expression levels of the reprogramming genes *DPPA2*, *LIN28*, *OCT4*, *HOXB3*, and *HOXB4* were similarly expressed in adult patients after UCBT and in iPS compared to UCB or adult HSPC. *NANOG*, *NES*, *SOX1*, and *SOX2* were upregulated in UCBT compared to iPS.

**Figure 2:**
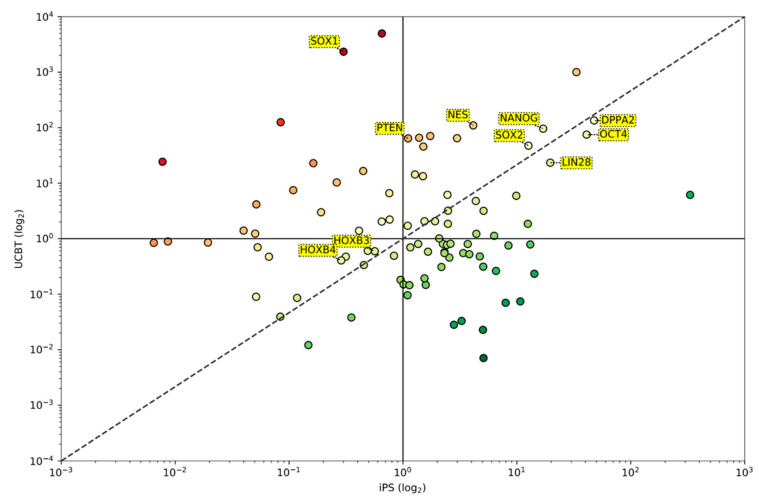
Log-log plot comparing the average expressions of genes in UCBT (y-axis) and iPS (x-axis). The dashed line represents the diagonal line (x = y), indicating equal average expression in UCBT and iPS. *DPPA2, NANOG, LIN28, OCT4, SOX2, HOXB3, and HOXB4* are expressed at similar levels as they lie close to the diagonal. The expression of many other genes, such as *PTEN, SOX1,* and *NES,* are significantly different. The color gradient from red to green is used to indicate genes upregulated in UCBT (red) and genes upregulated in iPS.

To further investigate the similarities and differences between iPS and UCBT, we analyzed the entire spectrum of 91 genes. Figure 2 shows the comparison between the average expression of the genes in UCBT (y-axis) and iPS (x-axis). We confirmed that *DPPA2*, *NANOG*, *LIN28*, *OCT4*, *SOX2*, *HOXB3*, and *HOXB4* were expressed at similar levels as they lie close to the diagonal (x = y) where gene expression in UCBT cells was equal to that observed in iPS. However, there were remarkable differences in the expression pattern of many genes such as *PTEN*, *SOX1*, and *NES*, whose expressions were significantly higher in adults after UCBT cells than in iPS.

### 3.2. The Transcription of the Overexpressed Genes Is Confirmed by Protein Analysis

**Figure 3:**
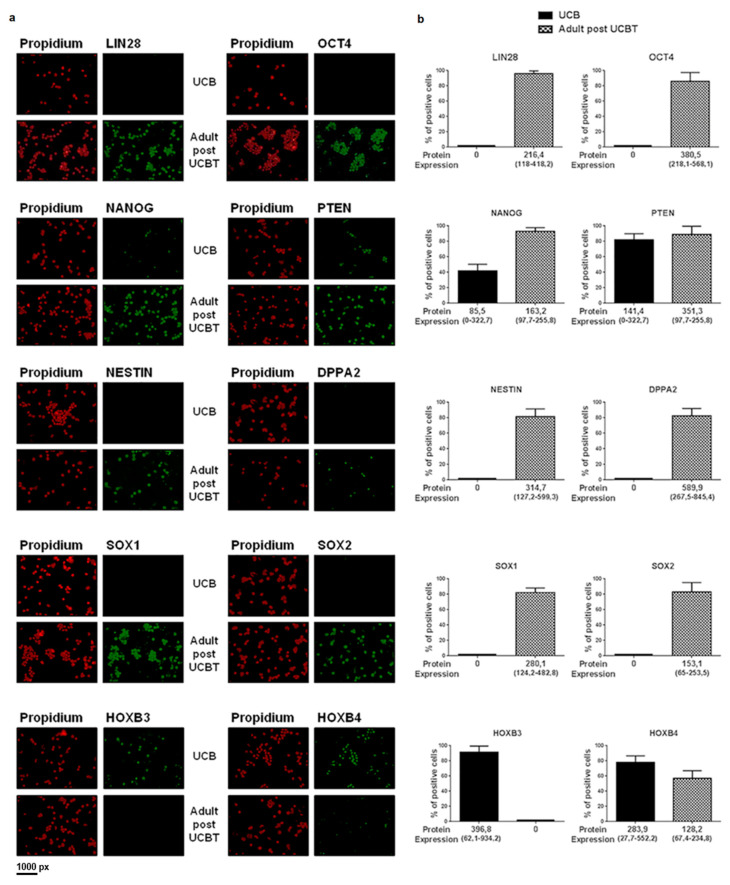
Panel **a**: Immunofluorescence using specific antibody (green signal) for the proteins indicated in top of the picture. Nuclei are marked in red. For each protein evaluated, the upper panels are referred to CD34+ cells isolated from UCB and the lower panels are referred to CD34+ cells isolated after UCBT. Panel **b**: For each protein indicated in top of the picture, the black column represents the percentage of CD34+ from UCB positive for the selected protein and the grey column the percentage of positive CD34+ from adult patients after UCBT. The mean value of protein expression in single cells and the range are indicated below the columns. Results showed that the number of cells expressing proteins is always higher in adults after UCBT compared to UCB. Nevertheless, the level of protein in single cells is always significantly lower in UCB (see median values and ranges reported under each graph).

Immunofluorescence analysis was carried out with specific antibodies (see Supplementary Table S1) recognizing proteins coded by DPPA2, LIN28, NANOG, NES, OCT4, PTEN, SOX1, SOX2, HOXB3, and HOXB4 in 69 samples analyzed by mRNA expression and in additional 44 samples with insufficient material for gene expression analysis. Figure 3 shows that the number of cells expressing the protein was always higher in adults after UCBT. In particular, for LIN28, OCT4, NESTIN, DPPA2, SOX1, and SOX2, no positive cells are detectable in UCB, whereas for adults after UCBT the percentage of positive cells ranges from 80% to 95%. Only for NANOG and PTEN, the percentage of positive cells was non-zero in UCB, being 40% and 80%, respectively. Nevertheless, the level of protein in single cells was always significantly lower in UCB (see median values and ranges reported for all proteins in Figure 3, panel b).

### 3.3. Sparse Multivariate Regularization Analysis 

To better capture the interplay among genes in the different groups (classes) of stem/progenitor cell sources, we performed a sparse multivariate analysis based on l_1_l_2FS_, a machine learning regularization method for variable selection (see Materials and Methods). We investigated the same questions as in the univariate analysis comparing UCB vs. adult HSPC, UCB vs. adult after UCBT, adult HSPC vs. adult and pediatric after HSCT, and adult after UCBT vs. adult and pediatric after HSCT (see Supplementary Table S1). For each comparison, l_1_l_2FS_ identified an optimal set of weights associated with each of the 91 genes. By design, l_1_l_2FS_ forced some of the weights to be exactly zero, therefore, selecting only those genes associated with a non-null weight (gene signature). These weights were used to build a linear classifier that was associated with a prediction accuracy and an MCC (Matthews Correlation Coefficient) score evaluating how well the selected genes were indeed able to discriminate the two classes. We recall that the accuracy is the proportion of correctly classified samples among the total number of cases examined; therefore, a good classifier has high accuracy, possibly about 100%. The MCC score ranges between −1 and +1. The greater MCC, the better the prediction with negative score marking below chance accuracy. The signatures were presented in the form of lists of genes ranked according to a frequency score (see Materials and Methods) and visualized by means of a heatmap plot. To assess the functional association among the selected genes, we presented an inferred gene network obtained with the STRING webtool where recurring instances of neighboring genes were used to infer the associations among the genes in the signature (see Materials and Methods). Finally, in order to functionally characterize the gene signatures, we performed an enrichment analysis in the Kyoto Encyclopedia of Genes and Genomes (KEGG) pathway database, using the online toolkit WebGestalt, obtaining the enriched pathways. 

#### 3.3.1. Only Six Genes Are Sufficient to Discriminate UCB from Adult

**Figure 4:**
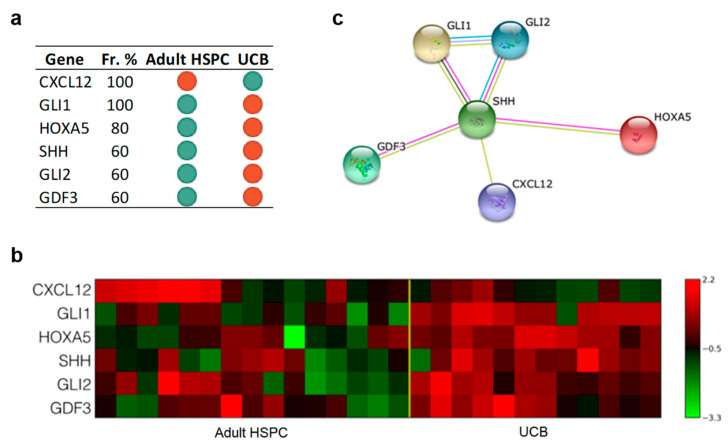
The results indicated that only six genes are sufficient to discriminate UCB from adult HSPC. Further, the Kyoto Encyclopedia of Genes and Genomes (KEGG) enrichment highlighted that Hedgehog pathway appears to be upregulated in UCB compared to adult HSPC. Panel **a**: Gene Signature. List of 6 gene symbols selected by the l_1_l_2FS_ procedure. For each gene, we report the corresponding frequency percentage score and the gene expression relative to the comparison between indicated groups: Green dots, downregulation; red dots, upregulation. Panel **b**: Heatmap associated with the 6 genes of the signature discriminating between adult HSPC (15 samples) and UCB (12 samples). The expression data for each gene has been scaled and is represented by pseudo-colors in the heatmap. Red color corresponds to a high level of expression and green color corresponds to a low level of expression as also shown in the color bar. Panel **c**: Association network of the genes in the signature between adult HSPC (15 samples) and UCB (12 samples) inferred by the STRING online tool. The identified connections are of five different types: Text mining (lime green), Experiments (magenta), Databases (Turquoise), Coexpression (Black), Homology (purple).

Multivariate analysis identified a combination of genes whose difference in expression can discriminate between UCB and adult CD34+ cells with an accuracy of 89% and an MCC of 0.8. The six genes are the following: *CXCL12*, *GLI1*, *HOXA5*, *SHH*, *GLI2*, and *GDF3*. The excellent prediction performance of our analysis was due to the homogeneity of the samples within each group. Figure 4 reports the selected genes (panel a) and the corresponding heatmap (panel b). Figure 4 panel c shows the network resulting from STRING analysis. Supplementary Table S3A reports the pathways we found enriched in KEGG. Among them, the Hedgehog pathway appears to be upregulated in HSPC from UCB compared to adult HSPC.

#### 3.3.2. The Set of the Expression Differences of Fifty-Two Genes Discriminates Cord Blood CD34+ Cells before and after Transplantation

**Figure 5:**
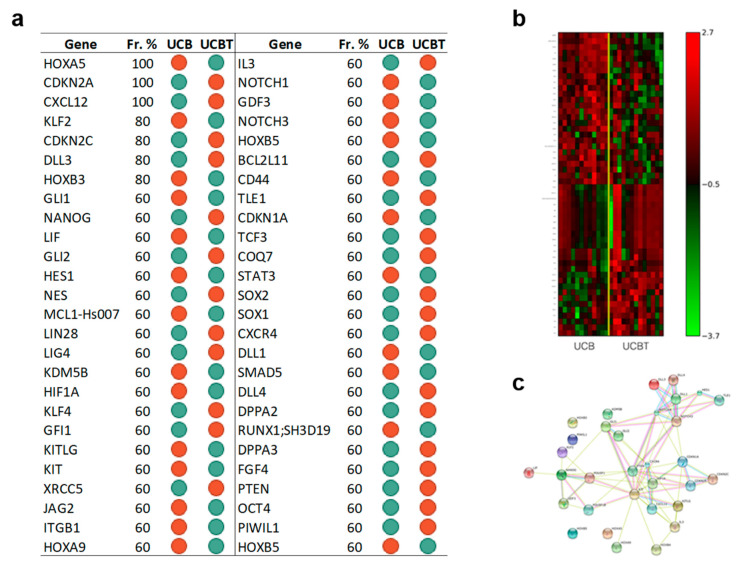
Data showed that a signature of 52 genes can discriminate UCB CD34+ cells before and after transplantation. Furthermore, KEGG analysis highlighted the involvement of NOTCH pathway and of genes regulating cytokine–cytokine receptor interaction. Panel **a**: Gene Signature. List of 52 gene symbols selected by the l_1_l_2FS_ procedure. For each gene, we report the corresponding frequency percentage score and the gene expression relative to the comparison between indicated groups: Green dots, downregulation; red dots, upregulation. Panel **b**: Heatmap associated with the 52 genes of the signature discriminating between UCB (12 samples) and UCBT (15 samples). The expression data for each gene has been scaled and is represented by pseudo-colors in the heatmap. Red color corresponds to a high level of expression and green color corresponds to a low level of expression as also shown in the color bar. Panel **c**: Association network of the genes in the signature between UCB (12 samples) and UCBT (15 samples) inferred by the STRING online tool. The identified connections are of five different types: Text mining (lime green), Experiments (magenta), Databases (Turquoise), Coexpression (Black), Homology (purple).

The set of the expression differences of fifty-two genes (Figure 5 panel a), including *NANOG*, *OCT4*, *PTEN*, *HOXB3*, and *HOXB4*, differentiated between CD34+ cells from UCB and after UCBT with an accuracy of 88% and an MCC value of 0.8. The corresponding heatmap is presented in Figure 5 panel b. Figure 5 panel c presents the associated gene network inferred by STRING. Supplementary Table S3B reports the pathways we found enriched in KEGG using the identified signature. In particular, among the 52 genes, the analysis extrapolated genes of the NOTCH pathway (*NOTCH3*, *NOTCH1*, *HES1*, *JAG2*) and genes involved in cytokine–cytokine receptor interaction.

#### 3.3.3. The Set of the Expression Differences of Sixty-Two Genes Discriminates Adult CD34+ Cells before and after Transplantation

**Figure 6:**
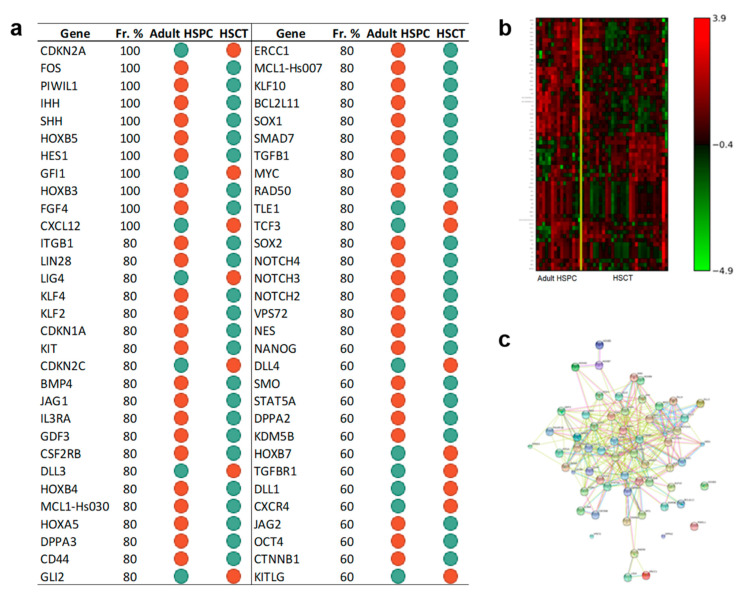
The results indicated that a signature of 62 genes can discriminate adult CD34+ cells before and after transplantation. Further, KEGG enrichment underlined the role of NOTCH and Hedgehog pathways. Panel **a**: Gene Signature. List of 62 gene symbols selected by the l_1_l_2FS_ procedure. For each gene, we report the corresponding frequency percentage score and the gene expression relative to the comparison between indicated groups: Green dots, downregulation; red dots, upregulation. Panel **b**: Heatmap associated with the 62 genes of the signature discriminating between adult HSPC (15 samples) and adult and pediatric after HSCT (29 samples). The expression data for each gene has been scaled and is represented by pseudo-colors in the heatmap. Red color corresponds to a high level of expression and green color corresponds to a low level of expression as also shown in the color bar. Panel **c**: Association network of the genes in the signature between adult HSPC (15 samples) and adult and pediatric after HSCT (29 samples) inferred by the STRING online tool. The identified connections are of five different types: Text mining (lime green), Experiments (magenta), Databases (Turquoise), Coexpression (Black), Homology (purple).

The same analysis was performed to compare CD34+ cells from adult donors and adult and pediatric patients transplanted with adult HSPC. The expression levels of 62 genes discriminated adult donor cells and cells collected after transplant in adult and pediatric patients with an accuracy of 88% and an MCC value of 0.7. The signature is reported in Figure 6 panel a. Interestingly, the selected genes included *NES*, *SOX1*, *SOX2*, *OCT4*, *DPPA2*, *NANOG*, and *LIN28*, all these genes were downregulated after transplant compared to the adult HSPC before transplant. The associated heatmap is shown in Figure 6 panel b. Figure 6 panel c presents the associated gene network inferred by STRING. Supplementary Table S3C reports the pathways we found enriched in KEGG using the identified signature. The analysis extrapolated genes of the NOTCH pathway (*JAG1*, *JAG2*, *NOTCH2*, *NOTCH3*, *NOTCH4*, *HES1*) and genes of the Hedgehog pathway (*GLI2*, *IHH*, *SHH*, *SMO*).

#### 3.3.4. After Transplantation Adult Versus Cord Blood CD34+ Cells Acquire a Divergent Transcriptional Asset; Reprogramming Genes Are a Relevant Part of Such Difference

**Figure 7:**
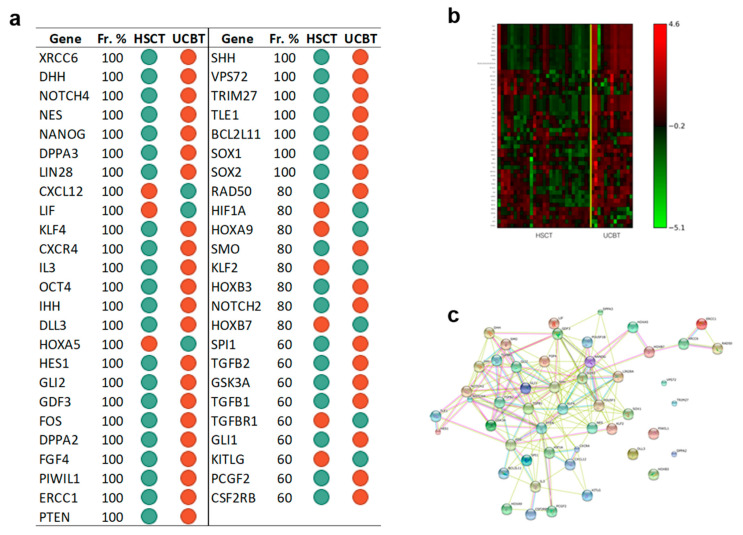
Data showed that CD34+ cells after transplantation acquire a divergent transcriptional asset based on the source of HSC: Adults HSPC or UCB. We found that genes involved in reprogramming and self-renewal are a relevant part of such difference. Furthermore, NOTCH and Hedgehog pathways were highlighted as relevant in KEGG analysis. Panel **a**: Gene Signature. List of 49 gene symbols selected by the l_1_l_2FS_ procedure [1,2]. For each gene we report, the corresponding frequency percentage score and the gene expression relative to the comparison between indicated groups: Green dots, downregulation; red dots, upregulation. Panel **b**: Heatmap associated with the 49 genes of the signature discriminating between adult after UCBT (13 samples) and adult and pediatric after HSCT (29 samples). The expression data for each gene has been scaled and is represented by pseudo-colors in the heatmap. Red color corresponds to a high level of expression and green color corresponds to a low level of expression as also shown in the color bar. Panel **c**: Association network of the genes in the signature between adult after UCBT (13 samples) and adult and pediatric after HSCT (29 samples) inferred by the STRING online tool. The identified connections are of five different types: Text mining (lime green), Experiments (magenta), Databases (Turquoise), Coexpression (Black), Homology (purple).

Finally, the comparison between UCBT and CD34+ after HSCT identified a set of expression differences of 49 genes including *NES*, *NANOG*, *LIN28*, *OCT4*, *DPPA2*, *SOX1*, *SOX2*, and *PTEN*, which is shown in Figure 7 panel a, together with its heatmap (Figure 7 panel b). This gene list distinguished patients according to the type of stem cells they received with an accuracy of 85% and an MCC value of 0.7. Figure 7 panel c presents the associated gene network inferred by STRING. Supplementary Table S3D reports the pathways we found enriched in KEGG using the identified signature. Among the identified genes, the analysis extrapolates genes of the NOTCH pathway (*HES1*, *DLL3*, *NOTCH2*, *NOTCH4*) and the Hedgehog pathway (*SHH*, *IHH, DHH*, *GLI1*, *GLI2*, *SMO*).

## 4. Discussion

Thousands and thousands of allogeneic transplants show that, in successful transplantations, the hematopoiesis is fully reconstituted and the bone marrow is morphologically similar to that of normal healthy donors [24]. The ultimate goal of HSPC is to ensure and maintain normal peripheral blood counts; this goal holds true when HSC are placed in a new body. The whole system is tightly regulated as witnessed by the stabilization of CD34+ cell frequency in the bone marrow within normal pre-transplantation levels irrespectively of the number of CD34+ cells infused and whether the graft is UCB or adult HSPC. Thus, so far, there were sufficient reasons to consider that in the recipient, after a short period of increased cell proliferation during the phase of reconstitution, the transplanted hematopoietic cells reproduce exactly the same picture of donor bone marrow. This may be intuitive for a transplant of adult bone marrow but not necessarily for cord blood. It has been assumed that cord blood cells after being transplanted into an adult recipient make a transition and become adult bone marrow. However, this question has not been addressed. The results of our study contribute to give a partial answer to those questions and document that after transplant, HSPC undergo a profound change in their transcriptional asset while, functionally, remaining adherent to the commitment to hematopoietic lineage. There may also be unknown mechanisms in the bone marrow microenvironment that prevent downregulation of somatic gene expression program [25]. Previous studies aimed to explore the function of single genes involved in self-renewal utilized constitutional overexpression or knock-out or silencing of selected genes. An elegant example of such experimental approaches was to enhance in-vivo the HSC proliferative potential by generating HOXB4-Transduced hematopoietic stem cells [26]. An increased stem cell pool was obtained in mice transplanted with transduced cells but this was evident only when HSC were challenged in further transplants, whereas HOXB4-transduced HSPC did not expand above levels normally observed in unmanipulated mice, indicating that its overexpression does not override the regulatory mechanisms that maintain the HSPC pool size within normal limits. Our approach, while not providing a functional assay as above, discloses photography (snapshot) of how HSPC orchestrates multiple genes at the same time to reconstitute the hematopoietic system. Intriguingly, in univariate analysis, few genes including *DPPA2*, *NANOG*, *PTEN*, *OCT4*, *NES*, *SOX1*, *SOX2*, and *LIN28* emerge as significantly overexpressed in CD34+ cells obtained from several patients transplanted with UCB cells. Among these genes, *OCT4*, *SOX2*, *LIN28*, and *NANOG* are considered the master regulators of pluripotency in embryonic stem cells (ESC). They are proved critical for ESC maintenance and capable of reprogramming mature somatic cells [27,28,29,30]. This behavior is unique of transplanted UCB cells since this does not occur when examining CD34+ cells after adult HSC transplantation. Immunofluorescence assay confirmed the results of mRNA overexpression by showing high levels of the corresponding proteins. By measuring proteins at a single cell level, we found that the vast majority of BM CD34+ cells after UCBT show a clear overexpression of the proteins involved in self-renewal and re-programming, although with a high degree of variability from cell to cell thus reflecting the heterogeneity of CD34+ cell population. In native UCB CD34+ cells, the level of these proteins is too low to be detectable by this method. Only NANOG and PTEN are expressed in CD34+ cells from UCB but the number of positive cells and the amount of protein in each single cell are lower than in CD34+ cells from UCBT. When we found the overexpression of genes and proteins involved in reprogramming, we did not have a reference level of expression associated with specific functional properties; thus, we investigated gene expression in different iPS cell lines. In CD34+ cells after UCBT, genes considered crucial for re-programming (*NANOG*, *OCT4*, *SOX2*, *LIN28*) showed a pattern of expression nearly superimposable to iPS cells. The acquisition of such gene expression configuration by CD34+ cells after UCBT is a remarkable finding and represents, so far, a unique example of “spontaneous” expression of reprogramming genes by somatic cells in adult life. In this context, it might be interesting to refer to the high propensity of UCB cells to be reprogrammed to iPS by the transduction of only two factors such as OCT4 and SOX2 [9]. One could speculate that the slower recovery of peripheral blood values observed after UCBT could be also interpreted as a resistance to differentiation and maturation of UCB HSPC. Whatever the case, as clearly shown by the regression analysis comparing gene signatures from UCBT and iPS, the vast majority of genes are differentially expressed in the two cell types; thus, excluding that CD34+ cells after UCBT have become similar to iPS. At the same time, the transcriptional changes that HSPC undergo during transplantation may be informative to understand how HSPC govern their gene machinery in such a tremendous post-transplant proliferation, avoiding the risk of leukemic transformation. *PTEN*, for example, is lower in iPS as compared to CD34+ cells after UCBT. PTEN was reported not to be required for HSC self-renewal in fetal hematopoietic stem cells, however, it is critical in adult hematopoietic stem cell maintenance [31,32,33]. More intriguingly, its loss of function in adult HSPC can promote leukemogenesis over time [33] and represents one of the major obstacles in utilizing iPS cells (or ESC cells) for clinical purposes. Here, we found that *PTEN* is significantly upregulated after UCBT CD34+ cells compared to iPS cells. In contrast, *PTEN* does not significantly change after transplantation of adult HSC. Since it is evident that CD34+ cells after UCBT do not generate neoplasia, the present study may represent an initial platform to learn how CD34+ cells are orchestrating their gene expression by combining tremendous expansion and, at the same time, avoiding transformation. Perhaps, the most relevant finding of our study is that adult CD34+ cells after HSCT show overexpression of specific genes, which is consistent and remarkably different from the set of genes overexpressed after UCBT. In designing this study, we were convinced to have the possibility to identify the genes overexpressed in order to achieve the hematopoietic reconstitution irrespective of the source of CD34+ cells used. Surprisingly, UCB and adult CD34+ cells seem to utilize different transcriptional machinery to cope with this challenge. It remains unexplored whether UCB CD34+ cells use a different transcriptional asset because they have to cope with extra expansion with respect to adult CD34+ cells or whether they can recruit genes that adult CD34+ cannot express anymore. There is another aspect that stands out. It has been difficult to “convince” ESC to become HSPC by transplanting ESC into irradiated mice [34]. In that model, it was only when the expression of genes such as HOXB4 was forced that transplanted ESC were capable to generate hematopoiesis [26]. However, in subsequent studies, HOXB4 has not functioned similarly in human ESCs since ectopic expression of HOXB4 did not affect repopulating capacity of hESC-derived cells [28]. Multivariate learning algorithm showed that four specific gene signatures can be obtained by comparing the four types of CD34+ cells; each signature generated by comparing the expression of genes in groups of two is characterized by an excellent prediction capability of the cell source and by an accuracy ranging from 80% to 90%.

## 5. Conclusions

Transplanted CD34+ cells considerably change their transcriptional asset when challenged with the need for hematopoietic reconstitution. This study places the question of understanding why to achieve hematopoietic reconstitution UCB HSPC choose a different transcriptional asset from adult HSPC including the recruitment of reprogramming genes and, at the same time, remain adherent to hematopoietic lineage. These results reveal undisclosed characteristics and potentialities of hematopoietic stem/progenitor cells and open a new area of research in transplantation biology.

## Supplementary Materials

The following are available online at https://www.mdpi.com/2077-0383/9/6/1670/s1, Table S1: Clinical characteristics of the patients and donors; Table S2: list of genes evaluated in the expression analysis; Table S3: Enrichment analysis performed in KEGG, using the online tool WebGestalt, of the following groups: UCB vs. Adult HSPC (Panel A), UCB vs. UCBT (Panel B), Adult HSPC vs. Adult and Pediatric HSCT (Panel C), and Adult UCBT vs. Adult and Pediatric HSCT (Panel D).
