# Supplementary material for: Transplantation Induces Profound Changes in the Transcriptional Asset of Hematopoietic Stem Cells: Identification of Specific Signatures Using Machine Learning Techniques
Source: J Clin Med. 2020 Jun 1;9(6):1670. doi: 10.3390/jcm9061670 (PMC7355574; doi:10.3390/jcm9061670)
Supplement: Supplementary file 1 [file jcm-09-01670-s001.pdf]

**Table S1. Clinical characteristics of the patients and donors**

| Group | UPN | Age (years) | Diagnosis | Type of transplant | Months from transplant |
|---|---|---|---|---|---|
| **Cord blood unit** | 1.01 | 0 | | | |
| | 1.02 | 0 | | | |
| | 1.03 | 0 | | | |
| | 1.04 | 0 | | | |
| | 1.05 | 0 | | | |
| | 1.06 | 0 | | | |
| | 1.07 | 0 | | | |
| | 1.08 | 0 | | | |
| | 1.09 | 0 | | | |
| | 1.10 | 0 | | | |
| | 1.11 | 0 | | | |
| | 1.12 | 0 | | | |
| **Adult donors** | 2.01 | 22 | | | |
| | 2.02 | 28 | | | |
| | 2.03 | 43 | | | |
| | 2.04 | 36 | | | |
| | 2.05 | 42 | | | |
| | 2.06 | 62 | | | |
| | 2.07 | 47 | | | |
| | 2.08 | 21 | | | |
| | 2.09 | 57 | | | |
| | 2.10 | 55 | | | |
| | 2.11 | 25 | | | |
| | 2.12 | 44 | | | |
| | 2.13 | 38 | | | |
| | 2.14 | 45 | | | |
| | 2.15 | 54 | | | |
| **Adult pts post UCBT** | 3.01 | 29 | AML | IB-UCBT | 60 |
| | 3.02 | 50 | AML | IB-UCBT | 48 |
| | 3.03 | 30 | ALL | IB-UCBT | 4 |

| | | | | | |
|---|---|---|---|---|---|
| | 3.04 | 50 | AML | IB-UCBT | 90 |
| | 3.05 | 44 | AML | IB-UCBT | 24 |
| | 3.06 | 22 | SAA | IB-UCBT | 5 |
| | 3.07 | 20 | SAA | IB-UCBT | 1 |
| | 3.08 | 18 | SAA | IB-UCBT | 3 |
| | 3.09 | 54 | MDS | IB-UCBT | 8 |
| | 3.10 | 55 | AML | IB-UCBT | 13 |
| | 3.11 | 32 | AML | IB-UCBT | 12 |
| | 3.12 | 18 | ALL | IB-UCBT | 5 |
| | 3.13 | 18 | AML | IB-UCBT | 1 |
| **Pts post adult HSC** | 4.01 | 36 | AML | PBSCT | 7 |
| | 4.02 | 62 | ALL | PBSCT | 3 |
| | 4.03 | 61 | CML | PBSCT | 2 |
| | 4.04 | 53 | AML | PBSCT | 1 |
| | 4.05 | 34 | AML | PBSCT | 18 |
| | 4.06 | 10 | ALL | BMT | 1 |

| **Group** | **UPN** | **Age (years)** | **Diagnosis** | **Type of transplant** | **Months from transplant** |
|---|---|---|---|---|---|
| **Pts post adult HSC** | 4.07 | 7 | FA | PBSCT | 2 |
| | 4.08 | 9 | ALL | PBSCT | 1 |
| | 4.09 | 17 | AML | PBSCT | 1 |
| | 4.10 | 17 | AML | PBSCT | 5 |
| | 4.11 | 10 | ALL | BMT | 5 |
| | 4.12 | 6 | Thal | PBSCT | 4 |
| | 4.13 | 13 | CML | PBSCT | 2 |
| | 4.14 | 2 | Thal | BMT | 3 |
| | 4.15 | 2 | Thal | BMT | 4 |
| | 4.16 | 3 | ALL | PBSCT | 1 |
| | 4.17 | 5 | ALL | PBSCT | 3 |
| | 4.18 | 15 | AML | PBSCT | 2 |
| | 4.19 | 8 | AML | PBSCT | 1 |
| | 4.20 | 5 | MDS | PBSCT | 1 |
| | 4.21 | 2 | AML | PBSCT | 1 |

| | | | | |
|---|---|---|---|---|
| 4.22 | 7 | AML | PBSCT | 6 |
| 4.23 | 15 | AML | PBSCT | 3 |
| 4.24 | 11 | ALL | PBSCT | 1 |
| 4.25 | 5 | AML | PBSCT | 1 |
| 4.26 | 6 | Thal | PBSCT | 1 |
| 4.27 | 2 | ALL | BMT | 6 |
| 4.28 | 10 | ALL | PBSCT | 3 |
| 4.29 | 2 | ALL | PBSCT | 1 |

**Table S2. List of analysed genes**

| Gene | Assay code | Antibodies for IF | Gene function |
|---|---|---|---|
| GUSB | Hs99999908_m1 | | Housekeeping gene |
| 18S | Hs99999901_s1 | | Housekeeping gene |
| ABL1 | Hs00245445_m1 | | Housekeeping gene |
| AXIN1 | Hs00394718_m1 | | Wnt signaling, beta-cateninin-mediated signaling |
| BCL2L11 | Hs00708019_s1 | | BCL-2 family members, initiator of apoptosis |
| BMI1 | Hs00180411_m1 | | Repressive activity of many genes, including Hox genes,chromatin remodelling |
| BMP4 | Hs00370078_m1 | | Resoderm induction |
| CD44 | Hs01075861_m1 | | Lymphocyte activation, recirculation and homing, and in hematopoiesis |
| CDKN1A | Hs00355782_m1 | | Interactor of p53/TP53, regulates proliferation in response to DNA damage |
| CDKN2A | Hs00923894_m1 | | Interactor of p53/TP53, regulates proliferation in response to DNA damage |
| CDKN2C | Hs00176227_m1 | | Controls cell cycle G1 progression, regulate spermatogenesis |
| COQ7 | Hs01029186_m1 | | Regulation of metabolism, lifespan determination |
| CSF2RB | Hs00166144_m1 | | Cell differentiation |
| CTNNB1 | Hs00355049_m1 | | Wnt pathway. Stem cell maintenance and proliferation |
| CXCL12 | Hs00171022_m1 | SDF-1 (P-159X) : sc-74271 | Embryogenesis, immune surveillance, inflammation response, tissue homeostasis |
| CXCR4 | Hs00607978_s1 | | Leukocyte trafficking, regulation of innate and adaptive immunity |
| DHH | Hs00368306_m1 | | Morphogenesis and gonadal development |
| DLL1 | Hs00194509_m1 | | Differentiation of progenitor cells into the B-cell lineage, emergence of T cell NK cell precursors |
| DLL3 | Hs01085096_m1 | | Inhibitor of primary neurogenesis |
| DLL4 | Hs00184092_m1 | | Angiogenesis; inhibitor of endothelial cell proliferation and migration |
| DPPA2 | Hs00414521_g1 | DPPA2 (D-17): sc-69392 | Maintenance of stem cell pluripotency |
| DPPA3 | Hs01931905_g1 | | Maintenance of stem cell pluripotency |
| DPPA4 | Hs00216968_m1 | | Maintenance of stem cell pluripotency |
| ERCC1 | Hs01012158_m1 | | DNA repair |
| FGF4 | Hs00173564_m1 | | Embryonic development, cell growth, tissue repair, morphogenesis |
| FLT3 | Hs00174690_m1 | | Apoptosis, proliferation, and differentiation of hematopoietic cells |
| FOS | Hs00170630_m1 | | Cell proliferation, differentiation, and transformation |
| GDF3 | Hs00220998_m1 | | Cell growth and differentiation in both embryonic and adult tissues |
| GFI1 | Hs00382207_m1 | | Cell cycle regulator, hematopoietic differentiation |
| GLI1 | Hs00171790_m1 | | Stem cell proliferation |
| GLI2 | Hs01119974_m1 | | Embryogenesis |
| GSK3A | Hs00997938_m1 | | Cell division, proliferation, motility and survival |

| | | | |
|---|---|---|---|
| HES1 | Hs00172878_m1 | | Regulator of myogenesis |
| HIF1A | Hs00936371_m1 | | Regulator of cellular and systemic homeostatic response to hypoxia |
| HOXA5 | Hs00430330_m1 | | Expression, morphogenesis, and differentiation |
| HOXA7 | Hs00600844_m1 | | Cell development |
| HOXA9 | Hs00365956_m1 | | Cell development |
| HOXB3 | Hs00231127_m1 | HoxB3 (H-50): sc-28606 | Cell development |
| HOXB4 | Hs00256884_m1 | HoxB4 (A-15): sc-28607 | Stem cell expansion |
| HOXB5 | Hs00357820_m1 | | Lung and gut development |
| HOXB7 | Hs00270131_m1 | | Cell proliferation and differentiation |
| IHH | Hs01081801_m1 | | Cell growth, patterning and morphogenesis |
| IL3 | Hs00174117_m1 | | Cell differentiation |
| IL3RA | Hs00608141_m1 | | IL3 signaling, activation of STAT pathway |
| ITGA4 | Hs00168433_m1 | | Cell growth, division, survival, differentiation, migration and apoptosis |
| ITGB1 | Hs00559595_m1 | | Cell growth, division, survival, differentiation, migration and apoptosis |
| JAG1 | Hs01070032_m1 | | Notch signaling, cell-fate decisions during hematopoiesis |
| JAG2 | Hs00171432_m1 | | Embryonic development |
| KDM5B | Hs00366783_m1 | | DNA demethylation |
| KIT | Hs00174029_m1 | | Regulation of cell survival and proliferation, hematopoiesis, stem cell maintenance |
| KITLG | Hs00241497_m1 | | Cell survival and proliferation, hematopoiesis, stem cell maintenance |
| KLF10 | Hs00921811_m1 | | Regulation of cell growth |
| KLF2 | Hs00360439_g1 | | T-cell trafficking |
| KLF4 | Hs00358836_m1 | | Embryonic stem cells maintenance |
| LIF | Hs00171455_m1 | | Hematopoietic differentiation |
| LIG4 | Hs00172455_m1 | | DNA repair |
| LIN28 | Hs00702808_s1 | Lin28 (6D1F9 ): sc-293120 | Maintenance of ES cells |
| MCL1 | Hs00766187_m1 | | Regulation of apoptosis |
| MCL1 | Hs03043899_m1 | | Regulation of apoptosis |
| MSH2 DNA | Hs00953523_m1 | | Mismatch repair system |
| MYC | Hs00905030_m1 | | Cell cycle progression, apoptosis and cellular transformation |
| NANOG | Hs02387400_g1 | Nanog (H-2): sc-374103 | Early embryogenesis |
| NES | Hs00707120_s1 | Nestin (5C93): sc-71665 | Regulation of differentiation, proliferation and apoptosis, lymphoid maturation |
| NOTCH1 | Hs01062014_m1 | | Regulation of differentiation, proliferation and apoptosis |
| NOTCH2 | Hs01050719_m1 | | Regulation of differentiation, proliferation and apoptosis |
| NOTCH3 | Hs01128541_m1 | | Regulation of differentiation, proliferation and apoptosis |
| NOTCH4 | Hs00965889_m1 | | Regulation of differentiation, proliferation and apoptosis |

| | | | |
|---|---|---|---|
| PCGF2 | Hs00810639_m1 | | Negative regulation of the self-renewal activity of hematopoietic stem cells |
| PIWIL1 | Hs01041737_m1 | | Stem cell self-renewal, RNA silencing |
| POU5F1 | PHs00999634_Gh | Oct4 (C-10): sc-5279 | Early embryogenesis and embryonic stem cell pluripotency |
| PTEN | Hs02621230_s1 | PTEN (B-1): sc-133197 | Antagonizes the PI3K-AKT/PKB signaling pathway, neurogenesis |
| RAD50 | Hs00990023_m1 | | DNA repair |
| RB1 | Hs00153108_m1 | | Regulator of entry into cell division |
| RUNX1 | Hs01021970_m1 | | Development of normal hematopoiesis |
| SHH | Hs00179843_m1 | | Hedgehog pathway, embryogenesis, stem cell proliferation |
| SIRT1- | Hs01009006_m1 | | Ageing |
| SMAD5 | Hs00195437_m1 | | Inhibition of proliferation of hematopoietic progenitor cells |
| SMAD7 | Hs00998193_m1 | | Regulator of TGFb signaling, regulator cell cycle arrest in hematopoietic cells |
| SMARCC1 | Hs00268265_m1 | | Chromatin remodeling, neural development |
| SMO | Hs01090242_m1 | | Hedgehog pathway, embryogenesis, stem cell proliferation |
| SOX1 | Hs01057642_s1 | Sox-1 (L-20): sc-17317 | Embryonic development and in the determination of the cell fate |
| SOX2 | Hs01053049_s1 | Sox-2 Ab (S-15): sc-54517 | Early embryogenesis and for embryonic stem cell pluripotency |
| SPI1 | Hs02786711_m1 | | Myeloid and B-lymphoid cell development |
| STAT3 | Hs01047580_m1 | | Transcription factor, signal transducer , regulators of cell growth and apoptosis |
| STAT5A | Hs00234181_m1 | | Transcription factor, signal transducer , regulators of cell growth and apoptosis |
| TCF3 | Hs01012685_m1 | | Transcription factor regulating B and T lymphocyte development |
| TGFB1 | Hs00998133_m1 | | Proliferation, differentiation, adhesion, migration |
| TGFB2 | Hs00234244_m1 | | Proliferation, differentiation, adhesion, migration |
| TGFBR1 | Hs00610318_m1 | | Regulator of TGFb signaling, regulator cell cycle arrest in hematopoietic cells |
| TLE1 | Hs00270768_m1 | | Transcriptional corepressor, regulator of Wnt signal |
| TRIM27 | Hs00179059_m1 | | Enhancer of polycomb protein and repressor of gene transcription, differentiation of germ cells |
| VPS72 | Hs00195618_m1 | | DNA repair, acethylation, apoptosis, regulation of long term hematopoiesis |
| XRCC5 | Hs00221707_m1 | | DNA repair |
| XRCC6 | Hs00995282_g1 | | DNA repair |

**Table S3. Enrichment analysis performed in KEGG, using the online tool WebGestalt, of the following groups: UCB vs Adult HSC (Panel A), UCB vs UCBT (Panel B), Adult HSC vs Adult and Pediatric HSCT (Panel C), and Adult UCBT vs Adult and Pediatric HSCT (Panel D).**

**Panel 1A**

| KEGG Pathway | #Gene | Gene Symbol | AdjP |
|---|---|---|---|
| Basal cell carcinoma | 3 | GLI2, GLI1, SHH | 1.17e-07 |
| `Hedgehog signaling pathway | 3 | GLI2, GLI1, SHH | 1.24e-07 |
| Pathways in cancer | 3 | GLI2, GLI1, SHH | 2.53e-05 |

**Panel 1B**

| KEGG Pathway | #Gene | Gene Symbol | AdjP |
|---|---|---|---|
| Pathways in cancer | 12 | GLI2, HIF1A, PTEN, FGF4, ITGB1, KITLG, CDKN1A, RUNX1, KIT, STAT3, CDKN2A, GLI1, | 8.62e-14 |
| Notch signaling pathway | 7 | HES1, NOTCH1, NOTCH3, DLL3, JAG2, DLL4, DLL1 | 2.55e-12 |
| Cytokine-cytokine receptor interaction | 6 | KITLG, LIF, IL3, CXCL12, CXCR4, KIT | 1.38e-05 |
| Melanoma | 4 | CDKN1A, PTEN, FGF4, CDKN2A | 2.79e-05 |
| Dorso-ventral axis formation | 3 | NOTCH1, NOTCH2, PIWIL1 | 5.22e-05 |
| Hematopoietic cell lineage | 4 | KITLG, IL3, CD44, KIT | 6.59e-05 |
| Acute myeloid leukemia | 3 | RUNX1, KIT, STAT3 | 0.0007 |
| Glioma | 3 | CDKN1A, PTEN, CDKN2A | 0.0011 |

| | | | |
|---|---|---|---|
| **P53 signaling pathway** | **3** | **CDKN1A, PTEN, CDKN2A** | **0.0012** |
| **Chronic myeloid leukemia** | **3** | **RUNX1, CDKN1A, CDKN2A** | **0.0015** |
| **Leukocyte transendothelial migration** | **3** | **CXCL12, CXCR4, ITGB1** | **0.0060** |
| **Axon guidance** | **3** | **CXCL12, CXCR4, ITGB1** | **0.0075** |
| **Cell cycle** | **3** | **CDKN2C, CDN1A, CDN2A** | **0.0075** |
| **Jak-STAT signaling pathway** | **3** | **LIF, IL3, STAT3** | **0.0135** |
| **Chemokine signaling pathway** | **3** | **CXCL12, CXCR4, STAT3** | **0.0255** |

**Panel 1C**

| **KEGG Pathway** | **#Gene** | **Gene Symbol** | **AdjP** |
|---|---|---|---|
| **Pathways in cancer** | **16** | TGFB1, GLI2, CTNNB1, FGF4, MYC, SHH, ITGB1, SMO, KITLG, STAT5A, TGFBR1, BMP4, CDKN1A, FOS, KIT, CDKN2A | **3.16e-19** |
| **Notch signaling pathway** | **9** | HES1, DLL3, JAG1, DLL4, DLL1, NOTCH4, JAG2, NOTCH2, NOTCH3 | **4.32e-16** |
| **Chronic myeloid leukemia** | **6** | TGFB1, STST5A, TGFBR1, CDKN1A, MYC, CDKN2A | **2.65e-08** |
| **Cytokine-cytokine receptor interaction** | **8** | TGFB1, CSF2RB, CXCR4, KITLG, TGFBR1, CXCL12, KIT, IL3RA | **1.10e-07** |
| **Basal cell carcinoma** | **5** | BMP4, GLI2, CTNNB1, SHH, SMO | **4.27e-07** |
| **Hedgehog signaling pathway** | **5** | BMP4, GLI2, IHH, SHH, SMO | **4.67e-07** |
| **Colorectal cancer** | **5** | TGFB1, TGFBR1, CTNNB1, FOS, MYC | **7.86e-07** |
| **Dorso-ventral axis formation** | **4** | NOTCH3, NOTCH2, PIWIL1, NOTCH4 | **1.02e-06** |
| **TGF-beta signaling pathway** | 5 | TGFB1, TGFBR1, BMP4, MYC, SMAD7 | **3.67e-06** |
| **Cell cycle** | **5** | TGFB1, CDKN2C, CDKN1A, MYC, CDKN2A | **2.56e-05** |
| **Hematopoietic cell lineage** | **4** | KILG, CD44, KIT, IL3RA | **0.0002** |
| **Endocytosis** | **5** | TGFB1, TGFBR1, CXCR4, KIT, SMAD7 | **0.0003** |
| **Leukocyte transendothelial migration** | **4** | CXCR4, CXCL12, CTNNB1, ITGB1 | **0.0006** |

| | | | |
|---|---|---|---|
| Bladder cancer | 3 | CDKN1A, MYC, CDK2A | 0.0008 |
| MAPK signaling pathway | 5 | TGFB1, TGFBR1, FOS, FGF4, MYC | 0.0011 |
| Intestinal immune network for IgA production | 3 | TGFB1, CXCR4, CXCL12 | 0.0012 |
| Jak-STAT signaling pathway | 4 | CSF2RB, STAT5A, IL3RA, MYC | 0.0019 |
| Acute myeloid leukemia | 3 | STAT5A, KIT, MYC | 0.0020 |
| Melanoma | 3 | CDKN1A, FGF4, CDKN2A | 0.0027 |
| Leishmaniasis | 3 | TGFB1, FOS, ITGB1 | 0.0027 |
| Pancreatic cancer | 3 | TGFB1, TGFBR1, CDKN2A | 0.0027 |
| ErbB signaling pathway | 3 | STAT5A, CDKN1A, MYC | 0.0081 |
| Rheumatoid arthritis | 3 | TGFB1, CXCL12, FOS | 0.0081 |
| Chagas disease | 3 | TGFB1, TGFBR1, FOS | 0.0108 |
| Melanogenesis | 3 | KITLG, CTNNB1, KIT | 0.0108 |
| Osteoclast differentiation | 3 | TGFB1, TGFBR1, FOS | 0.0216 |
| Axon guidance | 3 | CXCR4, CXCL12, ITGB1 | 0.0216 |

**Panel 1D**

| KEGG Pathway | #Gene | Gene Symbol | AdjP |
|---|---|---|---|
| Pathways in cancer | 13 | TGFB1, GLI2, PTEN, HIF1A, FGF4, SHH, SMO, KITLG,TGFBR1, TGFB2, SPI1, FOS, GLI1 | 1.14e-15 |
| Cytokine-cytokine receptor interaction | 9 | CSF2RB, LIF, IL3, TGFB1, CXCR4, KITLG, TGFBR1, CXCL12, TGFB2 | 4.38e-10 |
| Hedgehog signaling pathway | 6 | DHH, GLI2, GLI1, IHH, SHH, SMO | 1.11e-09 |
| Notch signaling pathway | 4 | HES1, DLL3, NOTCH2, NOTCH4 | 5.50e-06 |
| Osteoclast differentiation | 5 | TGFB1, TGFBR1, TGFB2, SPI1, FOS | 8.10e-06 |
| Basal cell carcinoma | 4 | GLI2, GLI1, SHH, SMO | 1.04e-05 |
| Colorectal cancer | 4 | TGFB1, TGFBR1, TGFB2, FOS | 1.70e-05 |
| Dorso-ventral axis formation | 3 | NOTCH2, PIWIL1, NOTCH4 | 5.84e-05 |

| | | | |
|---|---|---|---|
| **Rheumatoid arthritis** | **4** | **TGFB1, CXCL12, TGFB2, FOS** | **7.94e-05** |
| **Chagas disease** | **4** | **TGFB1, TGFBR1, TGFB2, FOS** | **0.0001** |
| **MAPK signaling pathway** | **5** | **TGFB1, TGFBR1, TGFB2, FOS, FGF4** | **0.0003** |
| **Intestinal immune network for IgA production** | **3** | **TGFB1, CXCL12, CXCR4** | **0.0005** |
| **Renal cell carcinoma** | **3** | **TGFB1, TGFB2, HIF1A** | **0.0015** |
| **Pancreatic cancer** | **3** | **TGFB1, TGFBR1, TGFB2** | **0.0015** |
| **Chronic myeloid leukemia** | **3** | **TGFB1, TGFBR1, TGFB2** | **0.0017** |
| **Leishmaniasis** | **3** | **TGFB1, TGFB2, FOS** | **0.0017** |
| **Endocytosis** | **4** | **TGFB1, TGFBR1, CXCR4, TGFB2** | **0.0018** |
| **TGF-beta signaling pathway** | **3** | **TGFB1, TGFBR1, TGFB2** | **0.0020** |
| **Jak-STAT signaling pathway** | **3** | **CSF2RB, LIF, IL3** | **0.0160** |
| **Chemokine signaling pathway** | **3** | **CXCL12, CXCR4, GSK3A** | **0.0280** |

**Tables Legend**

**Table S1:** Clinical characteristics of the patients and donors. Abbreviations: ALL, acute lymphoid leukemia; THAL, thalassemia; AML, acute myeloid leukemia; MDS, myelodysplastic syndrome; FA, Fanconi anemia; CML, chronic myeloid leukemia; SAA, severe anaplastic anemia.

**Table S2:** list of genes evaluated in the expression analysis. First column: gene symbols; second column: identification codes of commercial assays used for RT-PCR experiments; third column: identification codes of antibodies uses for immunofluorescences; fourth column: gene function.

**Table S3:** Enrichment analysis performed in KEGG, using the online tool WebGestalt, of the following groups: umbilical cord blood unit (UCB) vs. Adult hematopoietic stem cells HSC (Panel A), UCB vs. patients after UCB transplant (UCBT) (Panel B), Adult HSC vs. Adult and Pediatric patients after HSC transplant (HSCT) (Panel C), and UCBT vs. Adult and Pediatric HSCT (Panel D). "#Gene" indicated the number of genes involved in the cited pathway among the selected 91; "AdjP" is the p value adjusted by the multiple test adjustment.
